# Evaluation of the Functional Outcome of Both Bone Forearm Fractures in the Pediatric Population With the Titanium Elastic Nailing System in a Tertiary Care Center

**DOI:** 10.7759/cureus.38747

**Published:** 2023-05-08

**Authors:** Tarun Kumar Somisetty, Hariprasad Seenappa, Vinod Kumar K., Arun H Shanthappa, Akshay P.

**Affiliations:** 1 Department of Orthopedics, Sri Devaraj Urs Medical College, Sri Devaraj Urs Academy of Higher Education and Research, Kolar, IND; 2 Department of Orthopedics, Sri Devaraj Urs Medical College, Sri Devaraj Urs Academy Of Higher Eduacation and Research, Kolar, IND

**Keywords:** tens, both bone fractures of forearm, minimally invasive procedure, pediatric fractures, forearm fractures

## Abstract

Background

Forearm fractures are one of the most common injuries in the pediatric population. Diaphyseal fractures of the forearm, in particular, are among the most common injuries treated in the pediatric population. The incidence of both bone forearm fractures has increased in the past decade.

Methodology

This is a hospital-based retrospective study conducted from June 2020 to December 2022 at R. L. Jalappa Hospital and Research Centre in the orthopedics department after obtaining clearance from the institutional ethics committee. Once inclusion and exclusion criteria are met, participants with both bone forearm fractures were treated with the Titanium Elastic Nailing System (TENS). Data were entered and analyzed using IBM Corp. Released 2011, IBM SPSS Statistics for Windows, Version 20.0 (IBM Corp, Armonk, NY, USA).

Results

Thirty patients were included in the study, with a mean age of 8.80 years. The majority were boys constituting 67% and girls constituting 33%. A road traffic accident was the mechanism of injury in the majority of patients (40%). The distal one-third forearm was the most common site fractured (63%). The mean flexion (active) at the elbow improved from 110°at at four weeks to 142° at 24 weeks. A restriction of about 23° in elbow extension at four weeks normalized to 0° at 24 weeks. The range of palmar flexion improved from 44°^ ^at four weeks to 68° at 24 weeks. The range of wrist dorsiflexion improved significantly over time from 46° at four weeks to 86° at 24 weeks. Complications such as delayed union and skin irritation were noted in two participants (6%).

Conclusions

Both bone forearm fractures treated with TENS have shown good results in terms of bony union and functional outcomes with the least complications.

## Introduction

The forearm has a complex anatomical architecture consisting of two parallel, mobile bones providing a stable link between the wrist and elbow. Diaphyseal fractures of the radius and ulna, commonly referred to as both bone forearm fracture, is one of the most common fractures among children, accounting for about 32% of all fractures [1,2]. As it serves as the origin of several muscles of the hand, restoration of forearm rotation, elbow and wrist movements, and grip strength following the fracture is facilitated by anatomic reduction and internal fixation. Historically, the majority of these fractures have been treated with nonoperative management relying on closed reduction and casting [3,4,5,6]. Recently, however, there has been a trend toward increased surgical management of these fractures to improve clinical outcomes. This trend is attributed to the complications of conservative management such as loss of mobility and re-displacement. Several authors have reported that internal fixation of diaphyseal forearm fractures has resulted in high union rates and acceptable forearm rotation [7,8,9,10,11,12,13].

The severity of the fracture ranges through a continuum from plastic deformity to significant displacement. The management of these fractures depends on the age, type of fracture, and extent of fracture displacement. Surgical treatment options include both rigid plate fixation and Titanium Elastic Nailing System (TENS) [8,9]. Recently, there has been an increased interest in determining patient-reported functional outcomes. A few reports have shown that Disabilities of the Arm, Shoulder, and Hand (DASH) and Short Musculoskeletal Function Assessment (SMFA) questionnaires correlated well with reduced range of motion of wrist and forearm. In this context, we intend to assess functional outcomes in children with both bone fractures of the forearm undergoing open reduction and internal fixation using a validated functional outcome instrument [11,12,13].

## Materials and methods

A hospital-based retrospective study was carried out in a tertiary care center between June 2020 and December 2022. Study participants included pediatric patients <16 years of age and presented to the orthopedics department with both bone fractures of the forearm managed surgically by TENS. Patients having compound fracture or pathological fracture, treated with casting before surgery, and any fracture for which definitive intervention was not taken within a month after the event occurred (neglected) both diaphyseal bone fractures were excluded from the analysis.

The sample size for the study was calculated to estimate the functional outcomes in these patients using the visual analog scale (VAS) and the DASH scoring system. Assuming an alpha error of 5%, the expected standard deviation of DASH scores to be 10, and desired precision of 4, the sample size was calculated to be 30 subjects by using t-distribution.

Patients who met inclusion and exclusion criteria were included in the study to determine the rate of union, functional outcome, and complications in forearm fractures in children treated with titanium elastic intramedullary nails. A total of 30 patients underwent TENS using the standard surgical procedure. Following surgery, these patients with comminuted fractures were immobilized in an above-elbow splint for four weeks, following which mobilization exercises were started gradually. The subjects were evaluated with VAS for assessment of pain and DASH [14] score to assess functional improvement preoperatively, immediately postoperative period, and every four weeks intervals up to 12 weeks and at 24 weeks. The range of motion and fracture nonunion were also assessed during the follow-up OPD visits. All these recorded data were taken up from the hospital database and analyzed.

Data were entered and analyzed using IBM Corp. Released 2011, IBM SPSS Statistics for Windows, Version 20.0 (IBM Corp, Armonk, NY, USA). Baseline clinical and demographic characteristics were summarized using proportion. The range of motion in the elbow and radioulnar joints, VAS, and DASH scores were summarized using mean and standard deviation. The complications were also summarized using proportion. A *P*-value less than 0.05 was considered statistically significant.

## Results

The age of the participants ranged from three to 16 years, with a mean (SD) age of 8.8 (2.3) years. About 53% of the participants were aged between six and 10 years, 27% of the participants were aged between 11 and 16 years followed by 20% of participants who were aged between zero and five years. Of the total 30 patients, 20 were males (67%) and the remaining 10 were females (33%).

About 57% of the participants (*n *= 17) had a fracture on the left side and 43% of the participants (*n *= 13) had a fracture on the right side, as mentioned in Table 1.

**Table 1:** Description of the demographic data and side of injury in the study participants (n = 30). SD, standard deviation

Characteristics	Frequency
Age, mean (SD) (years)	8.80 (2.3)
Preschool (0-5 years), *n* (%)	6 (20)
School children (6-10 years), *n* (%)	16 (53)
Adolescents (11-16 years), *n* (%)	8 (27)
Sex	
Male, *n* (%)	20 (67)
Female, *n* (%)	10 (33)
Side of injury	
Left, *n* (%)	17 (57)
Right, *n* (%)	13 (43)

About 8 participants (27%) had a mid-diaphysis fracture, 3 participants (10%) had a fracture at the upper one-third, and the remaining 19 patients (63%) had a lower one-third fracture of the radius and ulna, as mentioned in Table 2.

**Table 2:** Location of the fracture site in the study participants (n = 30).

Fracture site	Frequency
Proximal one-third region, *n* (%)	3 (10)
Middle one-third region, *n* (%)	8 (27)
Distal one-third region, *n* (%)	19 (63)

The majority of the fractures were transverse 18 (60%), 8 (27%) were short oblique type, 3 (10%) were comminuted type, and 1 (3%) was segmental fracture type, as mentioned in Table 3.

**Table 3:** Fracture pattern distribution in the study participants (n = 30).

Fracture pattern	Frequency
Transverse fracture, *n* (%)	18 (60)
Short oblique fracture, *n* (%)	8 (27)
Comminuted fracture, *n* (%)	3 (10)
Segmental fracture, *n* (%)	1 (3)

A road traffic accident is a common cause of injury in the study population constituting 12 (40%), followed by fall while playing in 8 (27%), self-falls in 7 (23%), and fall from height in 3 (10%), as mentioned in Table 4.

**Table 4:** Mechanism of injury in the study participants (n = 30).

Mechanism	Frequency
Self-fall, *n* (%)	7 (23)
Fall from height, *n* (%)	3 (10)
Fall while playing, *n* (%)	8 (27)
Road traffic accident, *n* (%)	12 (40)

All 30 patients were retrospectively treated with TENS. There was a significant restriction in the movements at the elbow and radioulnar joints following the injury and fixation. The mean flexion (active) at the elbow improved from 110° at four weeks to 142° at 24 weeks. A restriction of about 23° in elbow extension at four weeks normalized to 0° at 24 weeks. The degree of pronation was more compromised than supination. However, both significantly improved over 24 weeks of the postoperative period. The range of palmar flexion improved from 44° at four weeks to 68° at 24 weeks. The range of wrist dorsiflexion improved significantly over time from 46° at four weeks to 86° at 24 weeks, as mentioned in Table 5.

**Table 5:** Description of the mean range of motion at elbow and radioulnar joints in the study participants (n = 30). SD, standard deviation

	4 weeks (mean ± SD)	8 weeks (mean ± SD)	12 weeks (mean ± SD)	24 weeks (mean ± SD)
Flexion at elbow (Active)	110 ± 32	122 ± 30	130 ± 30	142 ± 36
Extension at elbow (Active)	23 ± 6	18 ± 5	10 ± 5	1 ± 2
Flexion at elbow (Passive)	114 ± 34	125 ± 30	136 ± 32	148 ± 34
Extension at elbow (Passive)	18 ± 5	13 ± 4	7 ± 3	0 ± 1
Supination	68 ± 26	74 ± 23	79 ± 18	89 ± 12
Pronation	56 ± 22	64 ± 20	73 ± 20	88 ± 18
Palmar flexion	44 ± 18	55 ± 16	62 ± 16	68 ± 18
Wrist dorsiflexion	46 ± 20	65 ± 18	78 ±20	86 ± 18

In this study, the mean reported VAS score among the participants was 8.3 during the preoperative period. There was a moderate decrease in the VAS score during the immediate postoperative period, and the decrease in VAS scores over time was found to be statistically significant. The mean DASH score upon admission was 35.8, which significantly reduced to 10.2 over 24 weeks, and the decrease was statistically significant as mentioned in Table 6. 

**Table 6:** Description of VAS and DASH scores over the different follow-up time in the study participants (n = 30). VAS, visual analog scale; DASH, Disabilities of the Arm, Shoulder, and Hand

	VAS score, mean (SD)	DASH score, mean (SD)	Radiological union, *n* (%)	*P*-value
Preoperative	8.3 (1.3)	35.8 (22)	-	<0.001
Postoperative (immediate)	6.8 (1.6)	24.3 (23)	-	<0.001
4 weeks	3.5 (1.4)	16.9 (12.0)	0	<0.001
8 weeks	2.9 (1.1)	14.3 (11.7)	19 (64)	<0.001
12 weeks	2.1 (0.7)	12.6 (9.4)	24 (80)	<0.001
24 weeks	1.2 (0.5)	10.2 (7.6)	29 (96)	<0.001

Complications following TENS were found among 2 (6%) patients out of 30 patients. One patient each had delayed union and skin irritation following TENS, as mentioned in Table 7.

**Table 7:** Complications following TENS fixation in the study participants (n = 30). TENS, Titanium Elastic Nailing System

Complications	Frequency, *n* (%)
Delayed union	1 (3)
Skin irritation	1 (3)
Total	2 (6)

## Discussion

Both bone forearm fractures in the pediatric population were traditionally managed by closed reduction and immobilization using a cast. Operative options for internal fixation among children include plating, intramedullary nailing, and pins. The closed reduction has shown better postoperative recovery in terms of reduced postoperative pain, fewer complications, and increased range of motion [4,5,15]. Intramedullary nailing is considered superior to compression plating, as there is an increased risk of nonunion, infection, nerve injury, ischemic contracture, or radioulnar synostosis associated with plate fixation. Van der Reis et al. reported a short operative time, minimal soft tissue dissection, ease of hardware removal, and early mobilization in intramedullary fixation [16].

The results of our study are consistent with the clinical and radiological results of previous studies of Open Reduction and Internal Fixation (ORIF) of fracture of both the radius and ulna [8,16,17,18,19,20,21,22]. Participants in our study had a mean age of 8.8 years, ranging between 0 and 15 years, with 53% of patients aged between six and 10 years. Therefore, the majority of the patients were aged >5 years and needed surgical intervention more frequently than those <5 years. In our study, the majority of the patients were male (67%) as compared to female (33%). This may be because male children are more aggressive and frequently engage in outdoor sports. In our study, the incidence of fracture was almost similar between the left and right sides. However, Kc et al., in a study done in 2013, reported that the incidence of fracture is higher on the left side because the left side is usually nondominant and used as a protective function while the patients fall on the ground [21].

There was a significant improvement in the range of motion in the elbow and radioulnar joints at 12 weeks and 24 weeks following TENS. This finding is similar to the systematic review done by Westacott et al., which compared the functional outcomes following intramedullary nailing or plate and screw fixation of pediatric diaphyseal forearm fractures [18]. The improvement in range of motion was significant over time in patients who underwent intramedullary nailing. The overall complication rate in our study was 6%, which is similar to the rates described by Kc et al. [21]. According to criteria by Price et al., 90% showed excellent outcomes, 7% showed good outcomes, and 3% showed fair outcomes [23].

Limitations of this study

The limitation of this study is that only a small sample size was studied and the results may not be precise estimates due to the pediatric age group. However, all patients were ensured complete follow-up up to 24 weeks postsurgery. Another potential limitation is the technique of assessment of forearm rotation.

## Conclusions

On the basis of this study's findings, we concluded that TENS fixation of diaphyseal fractures of both bones of the forearm restores near-normal anatomy and range of motion with better postoperative pain. In conclusion, TENS is an effective and minimally invasive method for the fixation of forearm fractures with excellent results in terms of bony union and functional outcomes with minimal complications. Therefore, TENS can be used as one of the treatment modalities in the management of pediatric forearm fractures below 16 years of age.

## References

[1] Kasser JR (1992). Forearm fractures. Instr Course Lect.

[2] Hedström EM, Svensson O, Bergström U, Michno P (2010). Epidemiology of fractures in children and adolescents. Acta Orthop.

[3] Caruso G, Caldari E, Sturla FD (2021). Management of pediatric forearm fractures: what is the best therapeutic choice? A narrative review of the literature. Musculoskelet Surg.

[4] Alrashedan BS, Jawadi AH, Alsayegh SO (2018). Outcome of diaphyseal pediatric forearm fractures following non-surgical treatment in a Level I trauma center. Int J Health Sci (Qassim).

[5] Bowman EN, Mehlman CT, Lindsell CJ, Tamai J (2011). Nonoperative treatment of both-bone forearm shaft fractures in children: predictors of early radiographic failure. J Pediatr Orthop.

[6] Elia G, Blood T, Got C (2020). The management of pediatric open forearm fractures. J Hand Surg Am.

[7] Di Giacinto S, Pica G, Stasi A (2021). The challenge of the surgical treatment of paediatric distal radius/ forearm fracture: K wire vs plate fixation - outcomes assessment. Med Glas (Zenica).

[8] Kapila R, Sharma R, Chugh A, Goyal M (2016). Evaluation of clinical outcomes of management of paediatric bone forearm fractures using titanium elastic nailing system: a prospective study of 50 cases. J Clin Diagn Res.

[9] Wall L, O'Donnell JC, Schoenecker PL, Keeler KA, Dobbs MB, Luhmann SJ, Gordon JE (2012). Titanium elastic nailing radius and ulna fractures in adolescents. J Pediatr Orthop B.

[10] McAuliffe JA (1997). Forearm fixation. Hand Clin.

[11] Duncan R, Geissler W, Freeland AE (1992). Immediate internal fixation of open fractures of the diaphysis of the forearm.. J Orthop Trauma.

[12] Pace JL (2016). Pediatric and adolescent forearm fractures: current controversies and treatment recommendations. J Am Acad Orthop Surg.

[13] Duncan R, Geissler W, Freeland AE, Savoie FH (1992). Immediate internal fixation of open fractures of the diaphysis of the forearm. J Orthop Trauma.

[14] Gummesson C, Atroshi I, Ekdahl C (2003). The disabilities of the arm, shoulder and hand (DASH) outcome questionnaire: longitudinal construct validity and measuring self-rated health change after surgery. BMC Musculoskelet Disord.

[15] Dodge HS, Cady GW (1972). Treatment of fractures of the radius and ulna with compression plates.. J Bone Joint Surg Am.

[16] Van der Reis WL, Otsuka NY, Moroz P (1998). Intramedullary nailing versus plate fixation for unstable forearm fractures in children. J Pediatr Orthop.

[17] Surgeon CO (2009). Flexible intramedullary fixation of pediatric forearm fractures-report on twenty-one patients. Bahrain Medical Bulletin.

[18] Westacott DJ, Jordan RW, Cooke SJ (2012). Functional outcome following intramedullary nailing or plate and screw fixation of paediatric diaphyseal forearm fractures: a systematic review. J Child Orthop.

[19] Satpathy GK, Sahoo D, Gouri LV (2022). Management of both bone forearm fracture using titanium elastic nailing system in pediatric age group. Asian Journal of Medical Sciences.

[20] Qidwai SA (2001). Treatment of diaphyseal forearm fractures in children by intramedullary Kirschner wires. J Trauma Acute Care Surg.

[21] Kc KM, Rc DR, Acharya P, Sigdel A, Lamal DK, Dahal SC (2021). Functional outcomes of pediatric both bone fractures fixed with titanium elastic nails: a hospital based study. Eur J Med Sci.

[22] Garg NK, Ballal MS, Malek IA, Webster RA, Bruce CE (2008). Use of elastic stable intramedullary nailing for treating unstable forearm fractures in children. J Trauma.

[23] Price CT, Scott DS, Kurzner ME, Flynn JC (1990). Malunited forearm fractures in children. J Pediatr Orthop.

